# A Horizontal Magnetic Tweezers and Its Use for Studying Single DNA Molecules

**DOI:** 10.3390/mi9040188

**Published:** 2018-04-17

**Authors:** Roberto Fabian, Christopher Tyson, Pamela L. Tuma, Ian Pegg, Abhijit Sarkar

**Affiliations:** 1 Department of Physics and Vitreous State Laboratory, The Catholic University of America, Washington, DC 20064, USA; pegg@cua.edu (I.P.); SARKAR@cua.edu (A.S.); 2 Biomedical Engineering Department and Vitreous State Laboratory, The Catholic University of America, Washington, DC 20064, USA; 88tyson@cua.edu; 3 Department of Biology, The Catholic University of America, Washington, DC 20064, USA; tuma@cua.edu

**Keywords:** single molecule micromanipulation, magnetic tweezers, DNA overstretching transition

## Abstract

We report the development of a magnetic tweezers that can be used to micromanipulate single DNA molecules by applying picoNewton (pN)-scale forces in the horizontal plane. The resulting force–extension data from our experiments show high-resolution detection of changes in the DNA tether’s extension: ~0.5 pN in the force and <10 nm change in extension. We calibrate our instrument using multiple orthogonal techniques including the well-characterized DNA overstretching transition. We also quantify the repeatability of force and extension measurements, and present data on the behavior of the overstretching transition under varying salt conditions. The design and experimental protocols are described in detail, which should enable straightforward reproduction of the tweezers.

## 1. Introduction

In the past 25 years, many new techniques for the micromanipulation of single DNA molecules and DNA-protein complexes have been developed. These allow molecular-level measurements of the elasticity of single DNA molecules through direct mechanical stretching and twisting studies as well as the exquisite step-by-step dissection of DNA-protein interactions. Such experiments have resulted in numerous insights into the role of DNA in the genetic processes it participates in, for example DNA replication [1], DNA transcription [2], and DNA recombination [3], all at the single molecule level. These data are difficult to obtain using standard ensemble-based biochemical and biophysical techniques [4].

Magnetic tweezers have emerged as a powerful technique for studying DNA and DNA-protein interactions at a single molecule level. Magnetic tweezers are uniquely suited for studying the mechanical response of biopolymers under controlled forces—such experiments are said to be performed in the constant force ensemble in which the DNA’s thermally-averaged extension is measured as a function of externally-generated forces. Technical advances now allow applied forces in the 0.1–100 pN range and extensions down to angstroms to be reliably measured (although typical setups are limited to detecting extension changes in the order of 1–10 nm) [5,6,7,8,9,10,11,12]. In addition to a pulling force, the DNA’s twist (or, more generally, linking number) [13] can also be modulated or, in a fixed-torque assay, direct, simultaneous and independent control of forces and torques on the DNA is also possible [14,15,16]. Other progress includes magnetic micro-manipulation of confined DNA [17], use of proteins as tethers (instead of DNA) [18], and a portable tweezers setup [19]. 

In typical magnetic tweezers, one end of the DNA is directly attached to the surface of the sample cell while the other end is attached to a micron sized superparamagnetic bead [20]. Above the sample cell are permanent magnets, typically 2 bar magnets, that generate a force that pulls the DNA in the vertical plane. The strength of the pulling force can be adjusted by moving the magnets away or closer to the sample cell. This design is commonly known as a vertical magnetic tweezers. The advantages of this configuration include versatility and simplicity, the ease with which DNA topology can be modulated (by rotating the magnet to control twist or introduce plectonemes), and the relative ease with which tethered DNA can be located. Various schemes have been developed to measure the extension of the DNA molecule with ever-increasing precision, including techniques based on counting the diffraction rings from the tethered bead [11,21,22]. Instrument designs that compensate for sample cell drift—mechanical drift can affect extension measurements in long-duration (~1 h) experiments—have also been developed [23,24]. However, these advances have added complexity to vertical magnetic tweezers and required sophisticated calibration techniques to determine the relation between diffraction rings and DNA extension.

Another option is to generate the forces on DNA tethers along the focal plane [24,25,26,27,28]. For example, Yan et al. [25] developed a magnetic tweezers configuration in which one end of the DNA is tethered on a non-magnetic bead while the other end of the DNA is tethered to a superparamagnetic bead. The non-magnetic bead is held by a micropipette aspiration while the magnetic bead is suspended near a permanent bar magnet which applies magnetic forces on the magnetic bead. However, they used a sample cell which increases buffer evaporation and other external noise. Extension measurements in horizontal tweezers are, in principle, straightforwardly obtained by subtracting the position of the two beads. These, in turn, are computed using image processing to determine the intensity-weighted centroid coordinates of the two beads. An added benefit is that differential extension measurements allow passive drift cancellation, a feature which is utilized in optical tweezers and some magnetic tweezers through the use of fiducial markers [29].

Here, we describe an improved version of a horizontal magnetic tweezers design previously reported in ref. [30]. As reported here, the tweezers is relatively simple to implement and can apply forces in the range of 0.5 pN to 100 pN (and higher) on single linear DNA molecules connected to 2 superparamagnetic beads, one to each end. One bead is immobilized by biotin–streptavidin interactions to the surface of a rigid glass pipette; this bead is connected by the DNA tether to another superparamagnetic bead suspended in buffer near a bar magnet. The force on the suspended bead can be modulated by adjusting the bead–magnet distance. Key features of our design include: (i) replacing pipette aspiration by a rigid functionalized glass pipette coated with biotin; (ii) incorporating a sample cell with a single narrow open slit that minimizes buffer evaporation and pressure fluctuation at the open interfaces; (iii) integrating an inlet and outlet into the sample cell for buffer exchange; (iv) using a second micromanipulator to manipulate the functionalized rigid glass pipette independently; (v) attaching the pipette micromanipulator on a rail system firmly fixed to the microscope in order to increase stability; (vi) floating the sample cell stage on a motorized micromanipulator that allows easy adjustment of the DNA-bead construct and the magnet distance. As a result, we achieve high precision measurements—for extensions, <10 nm, and for forces, 0.5 pN. We calibrate our force–extension measurements using the DNA overstretching transition.

## 2. Materials and Methods

### 2.1. DNA End-Functionalization

The first step is to end-functionalize the DNA tether. In the experiments reported here, we use linear bacteriophage λ-DNA (N3011S, New England Biolabs, Ipswich, MA, USA) with sequence-specific 12 base polynucleotide overhangs: 3′-end overhang is gggcggcgaccg and 5′-end overhang is aggtcgccgccc. Following standard protocols [30,31], the 3′-end overhang of the λ-DNA is ligated to an oligomer with a sequence aggtcgccgcccBattggBattccBttccaBgtttaB (Integrated DNA Technologies, Coralville, IA, USA), where B represents the biotin attached to the oligomer. The 5′-end overhang of the λ-DNA is ligated to an oligomer with a sequence gggcggcgacctBattggBattccBttccaBgtttaB (Integrated DNA Technologies) where B again represents the biotin attached to the oligomer. Both ends of λ-DNA are thus able to bind to the superparamagnetic beads coated with streptavidin.

### 2.2. Surface-Functionalization of the Glass Pipette

The second step is to surface-functionalize the glass pipette by coating the pipette tip with biotins. The pipette is formed by drawing 1 mm, thick-walled, glass capillaries (1B100-6, World Precision Instruments, Sarasota, FL, USA) in a horizontal micropipette puller (Sutter Instruments P-97, Novato, CA, USA). The following settings are used in the P-97: heat = 701, pull = 125, velocity = 110, and time = 140, and result in rigid pipettes. The pipette tips are next inserted into a solution that contains 10 mg silane polyethylene glycol (PEG) biotin (PG2-BNSL-600-2K, NANOCS, New York, NY, USA) dissolved in 200 μL solution of 90% ethanol (E7023, Sigma Aldrich, St. Louis, MO, USA) in water and left for 30 min at room temperature.

### 2.3. Sample Cell Preparation

The third step is to prepare the sample cell. The sample cell is constructed using two number 0 coverslips of differing lengths which we call coverslip 1 (72198-20, Electron Microscopy Science, Hatfield, PA, USA) and coverslip 2 (72198-10, Electron Microscopy Science). In addition, a custom 3D-printed spacer (Watershed XC 11122, Proto Labs Inc., Maple Plain, MN, USA) and a 3 mm × 2 mm × 1 mm neodymium bar magnet (M0301, SUPERMAGNETMAN, Pelham, AL, USA) are also used. The coverslip 1 and coverslip 2 are glued on top and bottom of the spacer forming the ceiling and floor of the sample cell. The sides of the spacer form three of the four sides of the sample cell; one side is left open to allow insertion of the pipette. The spacer has an inlet and outlet for buffer exchange. The bar magnet is glued at the bottom of the coverslip 2. A clear room-temperature vulcanizing (RTV) silicone sealant (All Purpose 100% Adhesive Sealant, DAP, Baltimore, MD, USA) is used for gluing the two coverslips and the bar magnet. The overall dimension of the sample cell is 60 mm × 40 mm × 1 mm; however, the enclosed region is 6 mm × 4 mm × 1 mm, yielding a sample cell volume ~100 μL.

### 2.4. DNA-Bead Construct

**Figure 1:**
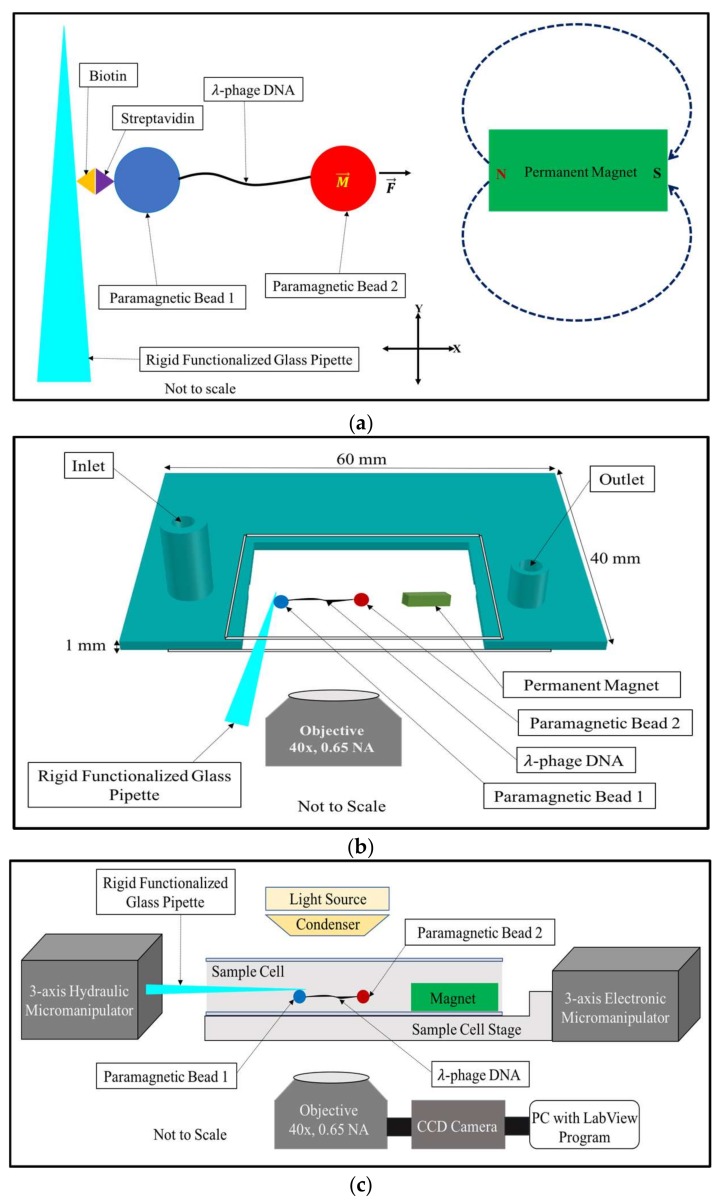
Basic principle of the horizontal magnetic tweezers: (**a**) the tweezers uses a rigid functionalized glass pipette to manipulate a bead attached to one end of a DNA tether and immobilized by biotin-streptavidin interactions to the surface of a rigid glass pipette. The other end of the tether is attached to a superparamagnetic bead suspended in buffer and placed at varying distances from a 3 mm × 2 mm × 1 mm bar magnet. Forces ranging from 0.5 pN to 100 pN (and higher) can be produced by moving the DNA-magnet distance from 2000 μm to 100 μm; (**b**) the design of the sample cell is shown. The sample cell has an inlet and an outlet for buffer exchange. The open side of the sample cell allows the insertion of the functionalized glass pipette used to capture and immobilize DNA-bead pairs; (**c**) a block diagram showing the layout of the horizontal magnetic tweezers.

The fourth step is to incubate in 1× Tris-EDTA (TE), 150 mM NaCl buffer (henceforth referred to as the “buffer”) at room temperature for about 10 min 5 µL end-functionalized DNA with 30 µL pre-washed 2.8 µm diameter superparamagnetic beads (11205D, Thermo Fisher Scientific, Waltham, MA, USA). After incubation, 5 μL of DNA-bead construct is introduced through the inlet of the sample cell—see Figure 1b. Once the DNA-bead construct settles to the floor of the sample cell, a Tygon tube (ACF00001, Saint-Gobain, Courbevoie, France) is connected to the inlet of the sample cell. The other end of this tube is connected to a syringe. The syringe is connected to a syringe pump (NE-1000, New Era Pump System, Farmingdale, NY, USA). Another Tygon tube (ACF00002, Saint-Gobain) is connected to the outlet of the sample cell, while the other end of this tube is connected to another syringe connected to a syringe pump (NE-1000, New Era Pump System). Prior to connecting the two tubes into the sample cell, all the tubes and syringes are filled with buffer. The pre-wash procedure for the superparamagnetic beads is described in detail in the supplementary materials.

### 2.5. Horizontal Magnetic Tweezers

The fifth step is to integrate the DNA-loaded sample cell and surface-functionalized glass pipette into the horizontal magnetic tweezers. The sample cell is mounted onto a 3D-printed sample cell holder made from a carbon-fiber reinforced plastic (CFRP). The sample cell holder is attached to a linear motorized stage system (X-MCB1, AB103B, XJOY3, Zaber, Vancouver, BC, Canada) which allows the sample cell to be moved in three axes. The motorized micromanipulator is controlled by a custom LabVIEW program (National Instruments, Austin, TX, USA) running on a Windows PC. The functionalized glass pipette is clamped into a custom-designed, two-piece, aluminum glass pipette holder; the holder is attached to a hydraulic, three-axis micromanipulator (MX630L S3432, Siskiyou Corporation, Grants Pass, OR, USA).

Next, after the clamped pipette is brought into focus, we carry out the following procedure to measure the distance between the magnet and the glass pipette: (i) using the motorized micromanipulator we bring the magnet to the pipette so that the face of the magnet touches the glass pipette; (ii) the magnet is moved parallel to the pipette so that the edge of the pipette is at the edge of the magnet; (iii) the controller for the motorized micromanipulator is zeroed, thus defining this point as the origin; (iv) the magnet is moved upward with respect to the pipette until the pipette rests on the floor of the sample cell and at bottom of the edge of the magnet; (v) repeat step (iii) above; (vi) the magnet is moved downward with respect to the pipette roughly 500 µm; (vii) repeat step (iii) above; (viii) the magnet is moved parallel to the pipette to position the edge of the pipette at the geometrical center of the rectangular magnet face, roughly 1000 mm from the magnet edge; (ix) repeat step (iii) above. At this point, the pipette reference point is at the geometrical center of the rectangular magnet face. Both syringe pumps connected to the inlet and the outlet, respectively, are activated to allow exchange of the buffer with fresh buffer.

The motorized stage system and the hydraulic micromanipulator are built around a Nikon Diaphot TMD inverted light microscope (Nikon, Tokyo, Japan) with a 40×, 0.65 NA, bright-field objective (Leica, Wetzlar, Germany). Imaging is performed using a Point Grey Grasshopper3 camera (GS3-U3-23S6M-C) with a frame rate of 120 Hz. A zoom lens (Edmund Optics, Barrington, NJ, USA) is also placed between the camera and the microscope objective. See the Supplementary Materials section for additional details about the design of the tweezers.

### 2.6. DNA Pulling Experiments

After completing the preceding steps, we are in a position to perform experiments. First, we must locate a DNA-tethered bead. Once a suitable candidate has been found, we move the magnet closer to the bead at a speed of 10 μm/s until we observe the DNA overstretching transition. This transition is very distinctive as the contour length of the DNA increases by 70%. This confirms that we have at least one DNA tethered to the suspended bead. Next, we move the magnet ~2 mm away from the tethered bead. Then, we start recording images using a custom Labview program.

The custom MatLab code [30] for data analysis uses a particle-tracking algorithm to detect the centroids of the two tethered beads in each frame and extract their coordinates. This allows computation of the tether’s extension, 〈z〉, as well as the transverse fluctuations of the suspended bead, $\langle \Delta y^{2}\rangle$. The applied force on the DNA is determined using the fluctuation–dissipation relation:

(1) $$F=\frac{k_{B}T\langle z\rangle }{\langle \Delta y^{2}\rangle }$$

Here, $k_{B}$ is the Boltzman’s constant, T is the absolute temperature of ~297 K, 〈z〉 is the DNA tether, and $\langle \Delta y^{2}\rangle$ is the variance in the transverse direction of the tethered bead.

To improve the precision of the force measurements, we average the force computed using Equation (1) at each positon/time interval from the magnet. This increases the force resolution as it smooths over fluctuations from mechanical and other a-thermal noise sources. We likewise average the extensions,〈z〉; however, it should be noted that even before this averaging step, each measurement of 〈z〉 has been spontaneously thermally averaged because the suspended, tethered bead is embedded in a thermal bath and the imaging system’s integration time is sufficient to allow proper sampling of the bead’s position from the relevant Boltzmann distribution; the additional averaging step corrects for a-thermally-generated noise.

We report three types of experiments. In the first set, we calibrate our magnetic tweezers by using it to determine the tether’s force–extension profile, including the force and extension associated with the overstretching transition, and then comparing it with the known results. Since we can reliably generate the overstretching transition in our system, we have an especially powerful way to establish the accuracy of our measurements. Force–extension measurements are taken by adjusting the distance between the supermagnetic bead suspended near the magnet and the magnet using the following protocol: all distances are measured from the magnet: 2 min each at 2 mm, 1.8 mm, 1.6 mm, 1.4 mm, 1.2 mm, 1.0 mm, 0.9 mm, and 0.8 mm; 1 min time each at 0.7 mm and 0.59 mm, 30 s each at 0.57 mm, 0.56 mm, 0.55 mm and 0.53 mm; 5 s each at 0.49 mm, 0.48 mm, 0.47 mm, 0.45 mm, 0.43 mm and 0.42 mm from the magnet. The position of the magnet is always adjusted at 10 μm/s.

The next set of measurements revolves around quantifying the resolution, i.e., precision or reproducibility, of the force and extension data. This is done by directly estimating the standard deviation, i.e., standard error (SE), in forces and extensions for experimental replications. We also used different copies of the magnets to analyze how much variability in the data is introduced by the choice of magnets.

The third set of experiments studied the extent of hysteresis in our force–extension data and especially the degree to which the overstretching transition is reversible. This can be done straightforwardly by moving the magnet closer to the tethered bead until the DNA’s contour length increases to ~70%, i.e., until we find the overstretching transition, then pause briefly, and then move the magnet away until its contour length decreases by 70%. Bead–magnet distances are adjusted at a speed of 10 μm/s. We also studied hysteric effects as a function of salt concentration by repeating these experiments with two different buffers.

Figure 1a shows the basic principles of the horizontal magnetic tweezers, Figure 1b shows the design of the sample cell, and Figure 1c shows the block diagram of the experimental setup.

## 3. Results and Discussion

**Figure 2:**
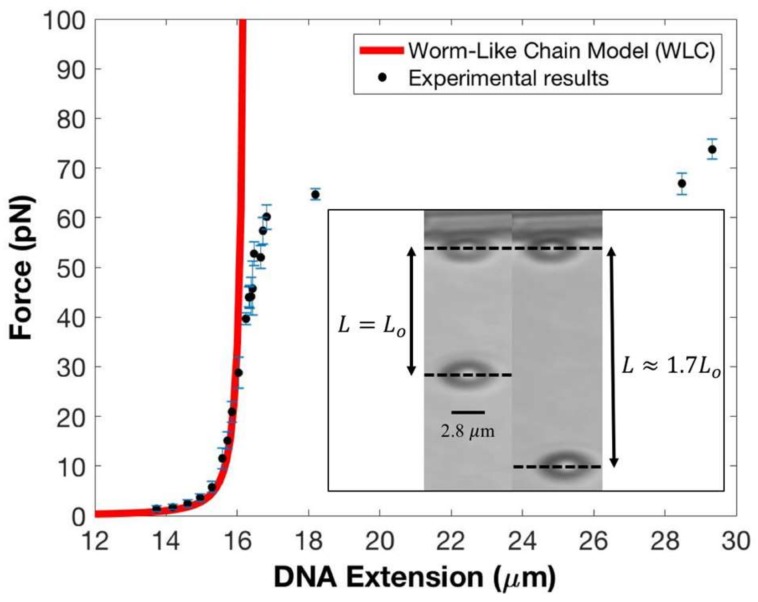
Experimental results of force vs DNA extension experiments on λ-DNA using the horizontal magnetic tweezers. Our data are represented by the solid black circles; corresponding standard errors in the forces are shown by blue lines. The solid red line represents the worm-like chain model for a single DNA molecule’s response to force, which agrees well with our data in the 1.0 pN to 40 pN force range. This corresponds to DNA extension from 13.7 μm to 16.2 μm. Between 40 pN to 60 pN our data begins to deviate to the right from the worm-like chain model. This is the region where the DNA is at is full contour length (around 25 pN) and then begins to stretch. At force range of 52.8 pN up to 60.1 pN with corresponding DNA extension of 16.5 μm up to 16.8 μm, our data further shifted to the right from the worm-like chain model. At force range of 64.7 pN up to 73.7 pN with corresponding DNA extension of 18.2 μm up to 29.3 μm, the DNA tether undergoes the overstretching transition with its zero-force contour length $L_{o}$ increasing by 70% of $L_{o}$. The inset shows two snapshots from our DNA extension experiments. From left to right, the first snapshot shows a single DNA with contour length of 16.4 μm with the tethered bead 550 μm from the magnet and corresponding to a force of 44.2 pN. The second snapshot shows a single DNA molecule in the overstretching transition with an extension of 28.5 μm, ~$1.7L_{o}$, at 430 μm from the magnet for a force of ~66.8 pN.

Figure 2 presents the results of a typical force versus extension measurement for a single DNA molecule plotted against the modified worm-like chain model (WLC) of DNA [32]:

(2) $$\frac{f_{z}b}{k_{B}T}=\frac{1}{4}{\left(1-\frac{\langle z\rangle }{L_{o}}+\frac{f_{z}}{K_{o}}\right)}^{-2}-\frac{1}{4}+\frac{\langle z\rangle }{L_{o}}-\frac{f_{z}}{K_{o}}$$

In Equation (2), $f_{z}$ is the applied force, $k_{B}$ is Boltzmann’s constant, T is the absolute temperature of ~297 K, b is the persistence length of 50 nm, $L_{o}$ is the contour length of 16.4 µm, $K_{o}$ is the elastic modulus of DNA which is ~1000 pN, and 〈z〉 is the DNA’s thermally-averaged end-to-end extension, which is the distance between the intensity-weighted centroids of the two beads—see Figure 2 caption. The solid black circles represent the experimental data while Equation (2) is plotted in red. The standard errors (SE) in the force are shown as blue lines; we did not include the S.E. of the DNA extension in Figure 2 because their magnitudes are too small compared to the magnitude of the S.E. of the force.

Our force–extension data matches well with the WLC model for forces up to ~40 pN, which covers the low-force non-linear entropic and high-force, linear, Hookean elastic response regimes. (Entropic elasticity refers to a range of forces in which the bending fluctuations in the DNA are suppressed but there is no change to its zero-force contour length; in the Hookean regime, however, the DNA’s backbone is stretched, thus altering the molecule’s contour length). Between ~40 pN to ~60 pN, we find an offset in our data with respect to the model’s predicted response. This is not unusual in this force range since the value of DNA’s elastic modulus, $K_{o}$, may change at these forces; additionally, the presence of DNA strand breaks (nicks) and whether single or both strands are tethered can also influence the elastic response.

At a force of ~65 pN, the tethered DNA undergoes the overstretching transition [24,33,34] with its contour length increasing by ~70%. The inset to Figure 2 shows two snapshots from an experiment with the left panel showing the tether at full extension, 〈z〉~16.4 μm with an applied force of 44.2 pN, and the right panel showing the tether in the canonical overstretched regime, with $\langle z\rangle \approx 1.7L_{o}$ at a force of 66.8 pN. Both the measured extension and the force at which the overstretching transition is initiated agree very well with established results. Moreover, the overall force–extension profile in this highly non-linear elastic regime is in very good agreement with known results. This provides an excellent calibration for our force and extension measurements as the overstretching transition has been observed using multiple, complementary experimental techniques and shows remarkable consistency between them [24,34,35]. Note that, by construction, the WLC model cannot reproduce the overstretching transition. The numerical values for magnet distances, corresponding forces, extensions, and standard errors displayed in Figure 2 are presented in Table 1. We point out that at forces starting at 50 pN, the superparamagnetic bead fluctuates at a frequency close to the Nyquist frequency [8,29], which may lead to sampling biases.

**Figure 3:**
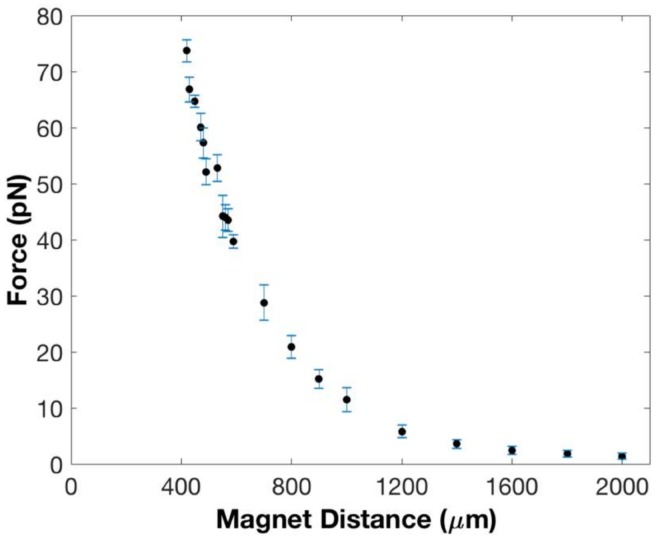
Magnetic force acting on a 2.8 μm superparamagnetic bead as a function of distance from the magnet. The solid black circles represent the force at each position from the magnet—see Table 1 columns 1 and 2. The blue lines represent the standard error in the forces—see Table 1 column 3.

We studied the reproducibility of our data by computing force and extension standard errors—see Table 1 columns 3 and 5—and also by assaying the impact on the data of using different copies of the magnet. As can be seen from columns 3 and 5 of Table 1, the precision of the force and extension measurements is consistent with other magnetic tweezers designs with forces of ~0.5 pN and extension changes of ~10 nm measured reproducibly using our instrument. Columns 1 and 2 of Table 1 have also been plotted in Figure 3.

**Figure 4:**
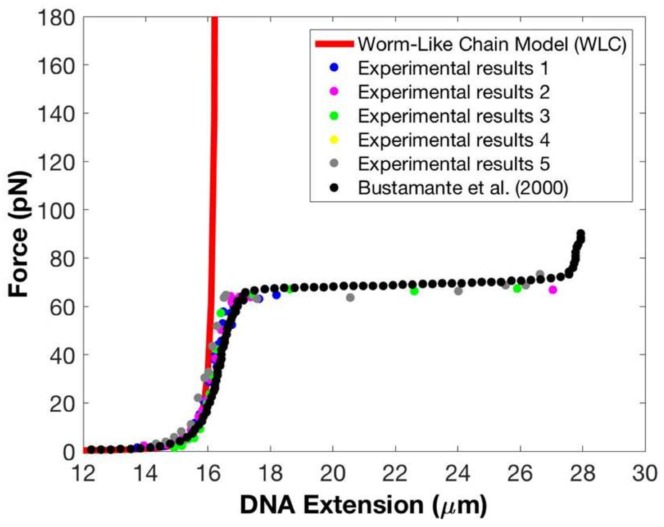
Replications of force–extension measurements using five different magnets and comparison to calibration data. The WLC is plotted as red solid line. The black solid circles represent the experimental results on λ-DNA using optical tweezers as presented in Strick et al. [11]. The remaining solid colored circles represent data from our experimental replications.

We also repeated force–extension experiments using five different copies of the magnet to check if we can reproduce our experimental results—see Figure 4. In addition to comparing our data with the predictions of the WLC model (solid red line), we also compared our results to the experimental data presented in Strick et al. [11] (solid black circles). Data from five experiments are displayed using five non-black or red solid colored circles. The results shown in Figure 4 provide evidence that force–extension data are not affected by using different copies of the magnet. We note that in spite of the inhomogeneity in magnets and superparamagnetic beads, over a wide range of magnet–bead distances, from 2 mm to 0.7 mm, we found a consistent relation between the magnet–bead distance and the applied force (and thus the tether’s extension). However, below distances of 0.7 mm, the magnet–bead distance at which a given force was produced varied with the magnet. Although the overstretching transition occurs at these distances, we found that the measured overstretching forces were highly consistent and independent of the magnet used.

Also evident in Figure 4 is the excellent agreement between the data from reference [11] obtained using optical tweezers to stretch λ-DNA, and our data across the entire force range.

**Figure 5:**
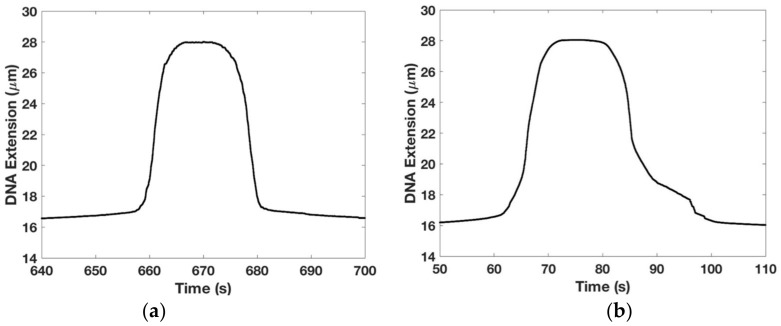
Hysteresis in DNA overstretching transition: (**a**) the extension-time graph of the DNA during a reversible overstretching transition. This experiment is done on 1× Tris-EDTA (TE) buffer with 150 mM NaCl at room temperature. Looking left to right, the extensional and contractile phases are symmetrical; (**b**) the extension-time graph of the DNA during a hysteretic overstretching transition. This experiment is done on 1× TE buffer in the absence of salt at room temperature. Looking left to right, the asymmetry between the extensional and contractile responses is clear visible. Bead-magnet distance are adjusted at a speed of 10 μm/s in (**a**,**b**).

We wanted to establish if our overstretching data were reversible and to study how the DNA’s elastic response was affected by the buffer’s salt concentration. The results are shown in Figure 5. Figure 5a shows that the DNA overstretching transition is reversible showing very little hysteresis, i.e., looking left to right, the extension profile is symmetrical with the contraction profile. This experiment was done by moving the magnet closer to the tethered DNA until it underwent the overstretching transition (length abruptly increased from $L_{o}$ (=16.4 μm) to $1.7L_{o}$ (=28 μm)) and then after a pause, moved the magnet away from the tethered DNA until its length decreased back to $L_{o}$—please see the Methods section for more details on the experimental protocol. We used 1× TE buffer with 150 mM NaCl with experiments performed at room temperature.

For comparison, Figure 5b shows hysteretic effects in the DNA overstretching transition when the experiment is repeated under otherwise identical conditions except 1× TE buffer with 0 mM NaCl is used. The asymmetry between the extensional and the contractile phases is marked and consistent with similar data from other groups [36,37]. This result may be interpreted as the presence of a large section of melted DNA beyond the overstretching force which prevents the two strands for re-annealing once the force is relaxed—see [36] for a detailed discussion.

## 4. Future Direction

We are currently carrying out single molecule micromanipulation experiments on DNA-compacting proteins. Looking ahead, we intend to modify the tweezers to incorporate fluorescence measurements, and improve our data analysis and validation approaches by using techniques described in refs. [38,39,40,41].

## Figures and Tables

**Table 1:** The values of the force, DNA extension and their corresponding standard error (S.E.) of the tethered DNA at certain distance from the magnet.

Magnet Distance (μm)	Force (pN)	S.E. of the Force (pN)	DNA Extension (μm)	S.E. of the DNA Extension (μm)
2000	1.4	0.56	13.7	0.056
1800	1.8	0.59	14.2	0.043
1600	2.4	0.73	14.6	0.032
1400	3.6	0.75	15.0	0.023
1200	5.8	1.15	15.4	0.016
1000	11.5	2.15	15.5	0.014
900	15.2	1.69	15.7	0.003
800	20.9	2.07	15.8	0.002
700	28.8	3.13	16.0	0.002
590	39.7	1.21	16.2	0.001
570	43.5	2.00	16.3	0.001
560	44.0	2.23	16.4	0.002
550	44.2	3.76	16.4	0.002
530	52.8	2.37	16.5	0.002
490	52.1	2.33	16.7	0.002
480	57.3	2.67	16.7	0.004
470	60.1	2.44	16.8	0.005
450	64.7	1.08	18.2	0.007
430	66.8	2.18	28.5	0.010
420	73.7	2.00	29.3	0.008

## Supplementary Materials

The following are available online at http://www.mdpi.com/2072-666X/9/4/188/s1, Horizontal Magnetic Tweezers Design, The prewash procedure for the superparamagnetic beads.

## References

[1] Maier B., Bensimon D., Croquette V. (2000). Replication by a single DNA polymerase of a stretched single-stranded DNA. Proc. Natl. Acad. Sci. USA.

[2] Finzi L., Gelles J. (1995). Measurement of lactose repressor-mediated loop formation and breakdown in single DNA molecule. Science.

[3] Bianco P.R., Brewer L.R., Corzett M., Balhorn R., Yeh Y., Kowalczykowski S.C., Baskin R.J. (2001). Processive translocation and DNA unwinding by individual RecBCD enzyme molecules. Nature.

[4] Strick T., Allemand J.F., Croquette V., Bensimon D. (2001). The manipulation of single biomolecules. Phys. Today.

[5] Daldrop P., Brutzer H., Huhle A., Kauert D.J., Seidel R. (2015). Extending the range for force calibration in magnetic tweezers. Biophys. J..

[6] Dulin D., Cui T.J., Cnossen J., Docter M.W., Lipert J., Dekker N.H. (2015). High spatiotemporal-resolution magnetic tweezers: Calibration and applications for DNA dynamics. Biophys. J..

[7] Kim K., Saleh O.A. (2009). A high-resolution magnetic tweezers for single-molecule measurements. Nucleic Acids Res..

[8] Lansdorp B.M., Tabrizi S.J., Dittmore A., Saleh O.A. (2013). A high-speed magnetic tweezer beyond 10,000 frames per second. Rev. Sci. Instrum..

[9] Haber B., Wirtz D. (2000). Magnetic tweezers for DNA micromanipulation. Rev. Sci. Instrum..

[10] Zacchia N.A., Valentine M.T. (2015). Design and optimization of arrays of neodymium iron boron-based magnets for magnetic tweezers applications. Rev. Sci. Instrum..

[11] Strick T., Allemand J., Croquette V., Bensimon D. (2000). Twisting and stretching single DNA molecules. Prog. Biophys. Mol. Biol..

[12] Kollmansberger P., Fabry B. (2007). High-force magnetic tweezers with force feedback for biological applications. Rev. Sci. Instrum..

[13] Strick T.R., Allemand J.F., Bensimon A., Croquette V. (1996). The elasticity of a single supercoiled DNA molecule. Science.

[14] Mosconi F., Allemand J.F., Croquette V. (2011). Soft magnetic tweezers: A proof of principle. Rev. Sci. Instrum..

[15] Janssen X.J.A., Lipfert J., Jager T., Daudey R., Beekman J., Dekker N.H. (2012). Electromagnetic torque tweezers: A versatile approach for the measurement of single-molecule twist and torque. Nano Lett..

[16] Lipfert J., Wiggin M., Kerssemakers J.W., Pedaci F., Dekker N.H. (2011). Freely orbiting magnetic tweezers to directly monitor changes in the twist of nucleic acids. Nat. Commun..

[17] Lin J., Persson F., Fritzsche J., Tegenfeldt J.O., Saleh A.O. (2012). Bandpass filtering of DNA elastic modes using confinement and tension. Biophys. J..

[18] You H., Wu J., Shao F., Yan J. (2015). Stability and kinetics of c-MYC promoter G-quadruplexes studied by single-molecule manipulation. J. Am. Chem. Soc..

[19] Yang Y., Lin J., Meschewski R., Watson E., Valentine M.T. (2011). Portable magnetic tweezers device enables visualization of the three-dimensional microscale deformation of soft biological materials. Biotechniques.

[20] Smith S.B., Finzi L., Bustamante C. (1992). Direct mechanical measurements of the elasticity of single DNA molecules by using magnetic beads. Science.

[21] Klaue D., Seidel R. (2009). Torsional stiffness of single superparamagnetic microspheres in an external magnetic field. Phys. Rev. Lett..

[22] Huhle A., Klaue D., Brutzer H., Daldrop P., Joo S., Otto O., Keyser U.F., Seidel R. (2015). Camera-based three-dimensional real-time particle tracking at kHz rates and Angstrom accuracy. Nat. Commun..

[23] Seol Y., Neuman K.C. (2011). Magnetic tweezers for single-molecule manipulation. Methods Mol. Biol..

[24] Chen H., Fu H., Zhu X., Cong P., Nakamura F., Yan J. (2011). Improved high-force magnetic tweezers for stretching and refolding of proteins and short DNA. Biophys. J..

[25] Yan J., Skoko D., Marko J.F. (2004). Near-field-magnetic-tweezer manipulation of single DNA molecules. Phys. Rev. E.

[26] Danilowicz C., Lee C.H., Kim K., Hatch K., Coljee V.W., Kleckner N., Prentiss M. (2009). Single molecule detection of direct, homologous, DNA/DNA pairing. Proc. Natl. Acad. Sci. USA.

[27] Sun B., Wei K., Zhang B., Zhang X., Dou S., Li M., Xi X. (2008). Impediment of E. coli UvrD by DNA-destabilizing force reveals a strained-inchworm mechanism of DNA unwinding. EMBO J..

[28] Schwarz F.W., Toth J., van Aelst K., Cui G., Clausing S., Szczelkun M.D., Seidel R. (2013). The helicase-like domains of type III restriction enzymes trigger long-range diffusion along DNA. Science.

[29] Moffitt J.R., Chemla Y.R., Smith S.B., Bustamante C. (2008). Recent advances in optical tweezers. Annu. Rev. Biochem..

[30] McAndrew C.P., Tyson C., Zischkau J., Mehl P., Tuma P.L., Pegg I.L., Sarkar A. (2016). Simple horizontal magnetic tweezers for micromanipulation of single DNA molecules and DNA-protein complexes. BioTechniques.

[31] Skoko D., Wong B., Johnson R.C., Marko J.F. (2004). Micromechanical analysis of the binding of DNA-bending proteins HMGB1, NHP6A, and HU reveals their ability to form highly stable DNA-protein complexes. Biochemistry.

[32] Marko J.F., Siggia E.D. (1995). Stretching DNA. Macromolecules.

[33] Cluzel P., Lebrun A., Heller C., Lavery R., Viovy J.L., Chatenay D., Caron F. (1996). DNA: An extensible molecule. Science.

[34] Smith S.B., Cui Y., Bustamante C. (1996). Overstretching B-DNA: The elastic response of individual double-stranded and single-stranded DNA molecules. Science.

[35] Paik D.H., Perkins T.T. (2011). Overstretching DNA at 65 pN does not require peeling from free ends or nicks. J. Am. Chem. Soc..

[36] Fu H., Chen H., Marko J.F., Yan J. (2010). Two distinct overstretched DNA states. Nucleic Acids Res..

[37] Wenner J.R., Williams M.C., Rouzina I., Bloomfield V.A. (2002). Salt dependence of the elasticity and overstretching transition of single DNA molecules. Biophys. J..

[38] Gosse C., Croquette V. (2002). Magnetic tweezers: Micromanipulation and force measurement at the molecular level. Biophys. J..

[39] Vilfan I.D., Lipfert J., Koster D.A., Lemay S.G., Dekker N.H., Hinterdorfer P., Oijen A. (2009). Magnetic tweezers for single-molecule experiments. Handbook of Single-Molecule Biophysics.

[40] Van Leonhout M.T., Kerssemakers J.W., De Vlaminck I., Dekker C. (2012). Non-bias-limited tracking of spherical particles, enabling nanometer resolution at low magnification. Biophys. J..

[41] Gross P., Laurens N., Oddershede L.B., Bockelmann U., Peterman E.J.G. (2011). Quantifying how DNA stretches, melts and changes twist under tension. Nat. Phys..

