# Supplementary material for: A Horizontal Magnetic Tweezers and Its Use for Studying Single DNA Molecules
Source: Micromachines (Basel). 2018 Apr 17;9(4):188. doi: 10.3390/mi9040188 (PMC6187538; doi:10.3390/mi9040188)
Supplement: Supplementary file 1 [file micromachines-09-00188-s001.docx]

A Horizontal Magnetic Tweezers and Its Use for Studying Single DNA Molecules

Roberto Fabian Jr., Christopher Tyson, Pamela L. Tuma, Ian Pegg and Abhijit Sarkar

Supplementary Materials

The pre-wash procedure for the paramagnetic beads.

(1) Take 30 μL of unwashed paramagnetic beads and place it in a 0.5 μL Eppendorf tube.

(2) Place a bar magnet at the bottom of the Eppendorf tube to separate the paramagnetic beads from the buffer.

(3) Remove the buffer by pipetting.

(4) Add 200 μL of 1X PBS into the paramagnetic beads and vortex the mixture and repeat steps (2) and (3).

(5) Add 200 μL of 1X TE buffer to the paramagnetic beads and vortex the mixture and repeat steps (2) and (3).

(6) Add 30 μL of 1X TE buffer to the paramagnetic beads and vortex the mixture.

Horizontal Magnetic Tweezers Design

The tweezers utilizes a Nikon Diaphot TMD (Nikon, Tokyo, Japan) inverted light microscope as the basis for the design. A 40× bright field objective (Leica, Wetzlar, Germany) with a 0.65 numerical aperture is used to image beads. A halogen lamp with condenser is used for illuminating the sample. Experiments are video recorded with frame rate of 120 Hz using a Point Grey Grasshopper3 camera (GS3-U3-23S6M-C), which connects to a Window PC using a USB3 connection. An adjustable zoom lens (Edmond Optics) is located between the microscope side port and the camera. The Point Grey camera interfaces with our own LabView Virtual Instrument program for monitoring and recording the experiments.

The components necessary for micromanipulation of single molecules are assembled upon the microscope as follows: Optical breadboard side platforms (Sutter Instruments, Novato, CA, USA) are mounted to the right and left of the microscope, while a pair of linear rails spans the two breadboards. The rigid functionalized glass pipette is clamped to a hydraulic micromanipulator (MX630L S3432, Siskiyou Corporation, Grants Pass, OR, USA) using a custom holder. The hydraulic micromanipulator is itself fixed to the middle of a custom-made rectangular aluminum fixture that is bolted to carriages affixed to the linear rails. The position of the carriages can be adjusted by sliding them on the rails. The hydraulic micromanipulator and associated assembly is positioned to the left of the microscope objective. The hydraulic micromanipulator is used to manually adjust the position of the rigid functionalized glass pipette during experiments.

The motorized stage (3× LSM Stage/3× XMCB, Controller/1× XJOY3/Cables, Zaber, Vancouver, BC, Canada) is bolted directly on to the right platform opposite the hydraulic micromanipulator and is characterized by excellent orthogonality of the motion control system, mechanical stability, and superior low-speed- control-velocities in the range of 200 nm per second are achievable. The stage system can be controlled manually with a joystick or from the PC. Figure 1 shows the photograph of the actual device that has been annotated to help identify the components-the color of the names in text at the bottom correspond to the colors of the outlines of the parts in the image.


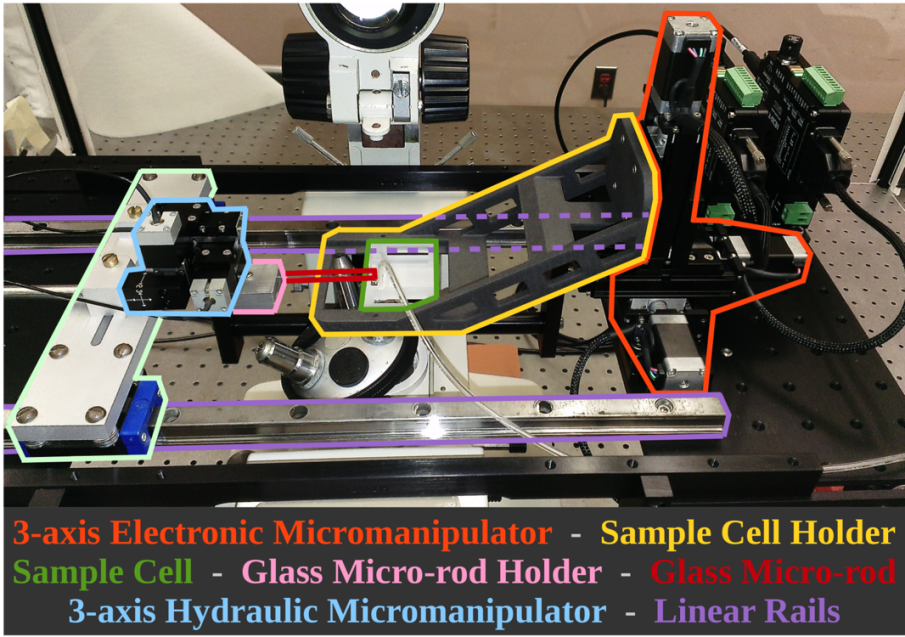


**Figure 1.** An annotated image shows the physical sizes and relationships of various components that comprise the horizontal magnetic tweezers. Names of items are colored to match the line that surrounds the item in the image.

© 2018 by the authors. Licensee MDPI, Basel, Switzerland. This article is an open access article distributed under the terms and conditions of the Creative Commons Attribution (CC BY) license (http://creativecommons.org/licenses/by/4.0/).
